# High Risk of Hepatocellular Carcinoma Development in Fibrotic Liver: Role of the Hippo-YAP/TAZ Signaling Pathway

**DOI:** 10.3390/ijms20030581

**Published:** 2019-01-29

**Authors:** Hyuk Moon, Kyungjoo Cho, Sunyeong Shin, Do Young Kim, Kwang-Hyub Han, Simon Weonsang Ro

**Affiliations:** 1 Yonsei Liver Center, Yonsei University College of Medicine, Seoul 03722, Korea; hmoon@yuhs.ac (H.M.); kyungjoo89@yuhs.ac (K.C.); SOONYOUNG94@yuhs.ac (S.S.); DYK1025@yuhs.ac (D.Y.K.); 2 Brain Korea 21 Project for Medical Science College of Medicine, Yonsei University, Seoul 03722, Korea; 3 Department of Internal Medicine, Yonsei University College of Medicine, Seoul 03722, Korea; 4 Institute of Gastroenterology, Yonsei University College of Medicine, Seoul 03722, Korea

**Keywords:** hepatocellular carcinoma, cirrhosis, regeneration, inflammation, cytokines, genetic instability, reactive oxygen species

## Abstract

Liver cancer is the fourth leading cause of cancer-related death globally, accounting for approximately 800,000 deaths annually. Hepatocellular carcinoma (HCC) is the most common type of liver cancer, making up about 80% of cases. Liver fibrosis and its end-stage disease, cirrhosis, are major risk factors for HCC. A fibrotic liver typically shows persistent hepatocyte death and compensatory regeneration, chronic inflammation, and an increase in reactive oxygen species, which collaboratively create a tumor-promoting microenvironment via inducing genetic alterations and chromosomal instability, and activating various oncogenic molecular signaling pathways. In this article, we review recent advances in fields of liver fibrosis and carcinogenesis, and consider several molecular signaling pathways that promote hepato-carcinogenesis under the microenvironment of liver fibrosis. In particular, we pay attention to emerging roles of the Hippo-YAP/TAZ signaling pathway in stromal activation, hepatic fibrosis, and liver cancer.

## 1. Introduction

Hepatocellular carcinoma (HCC) is the most common primary liver cancer in adults, leading to an increasing number of cancer-related deaths, especially in developing economies of Asia and Africa [1]. According to the World Health Organization (WHO), about 9.5 million deaths worldwide were related to cancer in 2018, among which 800,000 deaths were due to liver cancer, making it the fourth leading cause of cancer-related death (http://gco.iarc.fr/today/fact-sheets-cancers). Various risk factors for HCC development are known, such as hepatitis B virus infection, hepatitis C virus infection, alcohol abuse, intake of aflatoxin B1 (a fungal carcinogen present in food supplies associated with mutations in a tumor suppressor gene *TP53*), and metabolic syndrome [2].

**Figure 1:**
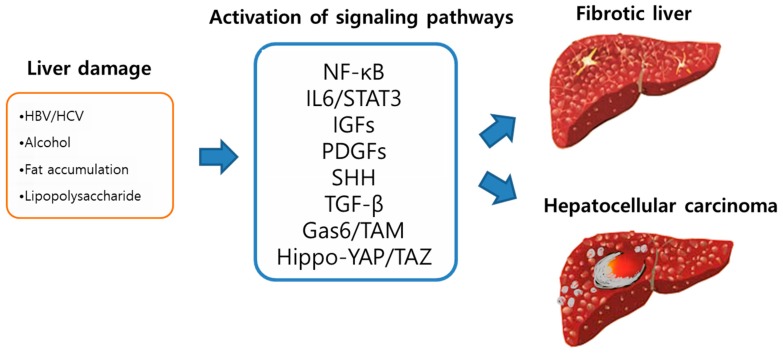
Schematic illustration of the mechanistic links between liver fibrosis and cancer. Persistent liver damage caused by viral infection, alcohol, fat, etc. lead to chronic inflammation and activation of various molecular signaling pathways, which contribute to both fibrogenesis and carcinogensis.

One of the most important features in liver cancer is that it is closely associated with liver fibrosis. Persistent liver damage caused by a variety of factors commonly leads to fibrosis in the liver. Hepatic fibrosis is accompanied in approximately 90% of patients with liver cancer, and the incidence rate of liver cancer within 5 years in patients with advanced liver fibrosis, or cirrhosis is as high as 5–30% [3,4]. Although a substantial increase in HCC development has been reported in cirrhotic patients as well as in animal models for hepatic fibrosis, the mechanism underlying enhanced hepato-carcinogenesis in hepatic fibrosis is not fully understood [5,6]. Several key features typically observed in fibrotic livers are suggested to create a pro-tumorigenic microenvironment, which are persistent hepatocyte death and compensatory regeneration, elevated inflammatory cytokines and growth factors, and an increase in reactive oxygen species. Recent years have seen a great advance in understanding the molecular mechanism linking liver fibrosis and cancer. Several molecular signaling pathways are found to be upregulated following liver damages and to promote hepatic fibrogenesis and liver cancer (Figure 1). Among the signaling pathways that include platelet-derived growth factor (PDGF), tumor growth factor beta (TGF-β), and sonic hedgehog (SHH) signaling pathways, we pay particular attention to the Hippo-YAP/TAZ signaling pathway in this review and introduce recent findings of new roles of YAP/TAZ signaling in hepatic fibrosis and cancer. 

## 2. Liver Fibrosis

A fibrotic liver exhibits major alterations in tissue architecture and function, which results from a chronic liver damage [7,8,9] induced by a variety of etiological factors including hepatitis viruses, alcohol and drug abuse, autoimmune disease and hereditary disorders of metabolism [10]. Most chronic liver diseases follow a rather common pathogenic pathway. A persistent hepatic injury induces a series of pathogenic processes from mild inflammation to more severe inflammation, to fibrosis, and finally to cirrhosis. Advanced fibrosis or cirrhosis is irreversible and associated with a significant morbidity and mortality, thus it is of a importance to understand the molecular mechanism underlying liver fibrosis and to prevent or decelerate the pathological process.

In normal liver, the extracellular matrix (ECM) provides structural support of surrounding cells including various molecules for cell adhesion, and allows cells to proliferate, grow, and migrate. It also enhances hepatic function and cell differentiation, and regulates cellular behavior and tissue formation [11]. Fibrosis, or excessive deposition of extracellular matrix components in hepatic tissue, however, compromises the structure and function of the tissue as signified by decreased macromolecular transfer between sinusoids and hepatocytes. Hepatic fibrosis leads to a distorted structure of sinusoids, which not only affects hepatocytes but also non-parenchymal cells such as hepatic stellate cells and myofibroblasts [12].

Activated ECM proteins, such as type I collagen, proteoglycans and glycoproteins, and hepatic stellate cells (HSC, also known as perisinusoidal cells or Ito cells) are the major components of fibrosis in the liver [13,14]. Continuous ECM protein accumulation leads to an increase in matrix stiffness and a change in the phenotype and function of hepatocytes, endothelial cells, and HSCs. In particular, hepatic fibrosis causes the loss of hepatocyte microvilli and accumulation of lipid droplets in hepatocytes as well as a decrease in endothelial fenestration [15,16,17]. Distorted vascular structure and decreased endothelial fenestration reduces transport of solutes from sinusoids to hepatocytes, further contributing to functional incompetence of hepatocytes. In addition, the alterations induced by the fibrotic microenvironment enhance a stimulus for HSCs to proliferate, activate, and migrate [12]. When activated, HSCs undergo differentiation into fibrogenic myofibroblasts, this produces α-smooth muscle actin (α-SMA), collagen type I and III, fibronectin, and etc. [7,18,19]. 

Hepatic function is significantly compromised in fibrotic microenvironment. For example, hepatocytes cultured on dishes coated with collagen type I show a rapid change in morphology along with the loss of hepatocyte-specific functions such as albumin and cytochrome P450 expression, while hepatocytes maintained on basement membrane proteins show preservation of the functions. As well, sinusoidal endothelial cells rapidly lose their fenestrae when cultured on a substratum of collagen type I [14]. Fibrosis can also affect cellular function indirectly via up-regulating various cytokines. These include transforming growth factor β (TGF-β), platelet derived growth factor (PDGF), hepatocyte growth factor (HGF), connective tissue growth factor (CTGF), tumor necrosis factor-α (TNF-α), basic fibroblast growth factor (bFGF), and vascular endothelial growth factor (VEGF). Overall, these architectural and functional changes provoke a positive feedback loop that further amplifies fibrogenic processes, resulting in progress to liver cirrhosis and organ failure [20].

Cirrhosis is caused by prolonged liver fibrosis [21], irreversibly destroying the liver structure and impairing the capability of liver to regenerate. The cirrhotic liver contains high concentrations of several cytokines or their effectors that influence hepatocyte fates. Defenestration and capillarization of sinusoidal endothelial cells are a major contributor to hepatic dysfunction in cirrhosis. In addition, activated Kupffer cells disrupt hepatocytes and facilitate the activation of HSCs. Repeated cycles of apoptosis and regeneration of hepatocytes promote the pathogenesis of cirrhosis [22].

For homeostasis during liver injury, ECM remodeling occurs with the balance between matrix metalloproteinases (MMPs) and their inhibitors, tissue inhibitors of matrix metalloproteinases (TIMPs). While an excessive ECM is down-regulated by MMPs (MMP-1, 2, 8 and 13), progressive fibrosis is associated with high expression levels of TIMPs (TIMP-1 and TIMP-2) [12,15,23]. Several studies reported that the down-regulation of TIMPs in HSCs could be an effective therapy for liver fibrosis [24,25]. 

## 3. Genetic Instability in Fibrotic Liver

An adult liver has a remarkable regenerating potential, as demonstrated by efficient restitution of a fully functional liver mass after acute 70% partial hepatectomy in mice and humans [26,27]. Liver fibrosis is a progressive tissue change with repeated death and compensatory regeneration of hepatocytes which is induced by chronic liver damage such as infection of hepatitis virus and consumption of alcohol. Increased cell turnovers in livers with chronic inflammation or fibrosis can create a pro-tumorigenic condition by increasing genetic mutations. Furthermore, damaged livers often reveal an increased level of reactive oxygen species (ROS), which further accelerate genetic mutation in the genome of hepatocytes by creating a mutagenic genetic environment (see below). Thus, persistent cellular death and compensatory regeneration in a fibrotic liver can lead to genetic instability, which predisposes liver parenchymal cells to oncogenesis [28]. Genetic instability in hepatocytes in the fibrotic liver can be achieved in several ways. 

First, regenerating hepatocytes in fibrotic livers undergo cycles of DNA replication required for cell divisions. DNA replication produces random genetic mutations such as base substitution, insertion, and deletion due to errors generated by DNA polymerase, which are imperfectly corrected by intracellular enzymes responsible for proofreading and repair. As the number of cell divisions increases during liver regeneration, mutations accumulate in the genome of hepatocytes, eventually leading to genetic alterations in cancer-related genes [29]. Tumorigenic processes can be initiated, for example, by an activating mutation in proto-oncogenes such as *RAS*, *SMO*, and *CTNNB1*, or a loss-of-function mutation in tumor suppressor genes such as *P53*, *Rb*, *p16^INK4A^*, etc. [30]. 

Secondly, excessive cell divisions in a fibrotic liver induce telomere shortening in hepatocytes, which increases the risk of tumorigenesis via chromosomal instability. In normal progenitor cells, a telomerase RNA component (TERC) maintains genomic integrity at the chromosome terminal region via telomere elongation. Because most mature hepatocytes lack the telomere-maintaining cellular machinery, continuous cell divisions can lead to substantial shortening of the telomere [31]. Telomere shortening in normal cells can trigger DNA damages as well as chromosomal instability, which can result in neoplastic transformation of premalignant cells. Interestingly, cancer cells acquire the ability to maintain telomere during tumor progression as an increased telomerase activity is found in 90% of human cancers [32,33]. How telomere shortening, which causes genetic instability and thus promotes transformation in exhausted hepatocytes, is recovered in cancer cells is currently a topic of intensive research [34,35]. 

Lastly, an increased production of ROS in liver fibrosis causes toxicity to cells and tissues by generating damaged proteins, lipids, and DNA [36,37]. Typically, elevated immune responses due to chronic infection of hepatitis virus produce an excessive level of ROS, which can further damage the liver. Notably, ROS cause various DNA adducts such as 4-oxo-2-alkenals, exocyclic etheno-DNA adducts, and 8-OHdG, which lead to base modifications in DNA [38]. Therefore, continuous ROS accumulation can significantly contribute to mutation in cancer-related genes and thus tumorigenesis [39]. Further, P53 cannot efficiently activate DNA repair mechanisms in the presence of a high level of ROS, enhancing genetic instability [40]. Supporting the mutagenic and carcinogenic effects, inhibition of ROS formation by antioxidants, butylated hydroxyanisole or N-acetylcysteine suppressed HCC development [41,42,43].

Of note, increased intracellular ROS activate various molecular signaling pathways that are closely related to tumorigenesis [44]. Excessive levels of ROS stimulate the TGF-β and NF-κB signaling pathways, which promote cancer initiation and progression (see below). Although numerous studies have shown a strong correlation between ROS and oncogenesis, there has been a controversy regarding their intracellular actions and precise roles during tumorigenesis, which is fueled by the lack of appropriate animal models to perform ROS measurements and to study ROS-mediated tumorigenesis in vivo.

## 4. Increased Secretion of Growth Factors and Cytokines

The liver is an immunologically complex organ, producing plasma proteins such as TNF-α, TGF-β and albumin [45] and soluble complement components which function as the innate immune defense [46,47]. The organ also contains a population of diverse resident immune cells [48,49,50]. In a healthy liver, metabolism, tissue remodeling, and exposures to microbial products induce inflammation to eliminate toxic substances, damaged cells, and hepatotropic pathogens [51]. In a chronically injured liver, activation of inflammatory cells and inflammatory responses aberrantly increase [52,53], leading to pathological inflammation and disruption of tissue homeostasis. Chronic inflammation induces changes in stroma [54], establishes pro-tumorigenic microenvironment [43,55], and activates various oncogenic molecular signaling pathways [56,57,58].

Many pro-inflammatory cytokines, including interleukin (IL)-1, IL-6, IL-17, and TNF-α are elevated in a chronically injured liver [59,60], leading to the activation of the nuclear factor kappa B (NF-κB) and the janus kinase (JAK)/signal transducer and activator of transcription factor (STAT) signaling pathways. Increased expression of PDGF, sonic hedgehog (SHH), and TGF-β1 are frequently observed in fibrotic livers caused by various etiological factors. These cytokines and growth factors play significant roles in hepatic fibrogenesis [61,62] and tumorigenesis [63,64].

### 4.1. Nuclear Factor Kappa B (NF-κB)

The NF-κB transcription factor is a key regulator inducing immune and inflammatory responses [65,66,67]. The most potent activators of NF-kB include inflammatory cytokines such as TNF-α or IL-1, as well as Toll-like receptors (TLRs) [68,69], which can also trigger cell proliferative signals through NF-Kb [70,71]. They activate NF-κB signaling via IκB kinase (IKK)-dependent phosphorylation and degradation of the κB inhibitor (IκB) proteins [72]. IKK consists of two catalytic subunits, IKKα and IKKβ, and regulatory elements, NEMO/IKKγ, which activate IKK mainly through IKKβ [73]. 

NF-κB is activated by various stimuli causing liver damages such as alcohol, excessive fat accumulation, hepatitis virus, and bacterial lipopolysaccharide (LPS). These hepatotoxic stimuli activate NF-κB-mediated pro-inflammatory responses leading to the transcription of hundreds of NF-κB target genes involved in the regulation of inflammation, immune responses and cell survival [72]. NF-κB also plays major roles in hepatic fibrosis, by regulating hepatocyte injury and triggering fibrogenic responses in the liver [72,74]. For example, LPS binds to Toll-like receptor-4 (TLR4) in HSC and activates the NF-κB signaling pathway, promoting survival and activation of HSC. Activated HSCs secrete chemokines that recruit and activate Kupffer cells, which are liver resident macrophages. Activated Kupffer cells then secrete TNF-α and IL-1 as well as TGF-β (see below), further enhancing HSC activation. Activation of NF-κB signaling in HSCs leads them to secrete various inflammatory cytokines and to induce quantitative and qualitative changes in the extracellular matrix [72]. TNF-α and IL-1 are frequently up-regulated in livers of chronic inflammation, which persistently activate the NF-kB signaling pathway in hepatocytes as well as non-parenchymal cells in the liver. 

NF-κB can exert a pro-tumorigenic effect via suppressing apoptosis during tumor development [75,76] through the positive regulation of anti-apoptotic factors, such as cIAPs, c-FLIP, and BclX [77]. Constitutive activation of NF-κB was frequently found in human HCC tissues compared with non-tumor tissues, and its activation was also verified in animal models of HCC [78]. Animal models with dysregulation of the NF-κB signaling pathway have shown spontaneous development of liver injury, inflammation, fibrosis and HCC, demonstrating that NF-κB acts as a mechanistic link between liver injury, inflammation, fibrosis and HCC [72,76]. Considering that patients with chronic liver inflammation and fibrosis exhibit activation of hepatic NF-κB signaling, it is of high significance and interest to investigate whether an increased incidence of HCC development in the patients can be attributed to NF-κB signaling [79].

### 4.2. IL6/STAT3 Signaling

IL-6 signals through a cytokine receptor complex that consists of the ligand-binding IL-6R and the signal-transducing component gp130 [80,81]. The IL6-bound hexameric signal transducing complex activates JAK tyrosine kinase, which phosphorylates and activates STAT3. Activated STAT3 dimers translocate to the nucleus and activate transcription of its target genes [82]. STAT3 inhibits the expression of mediators activating immune response against tumor cells [83] and has pro-mitogenic and anti-apoptotic effects on tumor cells [84]. 

Along with NF-κB signaling, IL6/STAT3 signaling is known as a major pro-inflammatory regulator in response to chronic liver damage. IL6, produced by activated Kupffer cells, is enriched in chronic liver inflammation, especially in non-alcoholic steato-hepatitis (NASH). A high STAT3 activity is also frequently observed in wounded livers, promoting survival and regeneration of hepatocytes. Upregulation of STAT3 signaling in Kupffer cells and HSC leads to subsequent pro-inflammatory and fibrogenic responses [79,83]. 

Expression of IL-6 and activity of STAT3 are found elevated in HCC [85]. STAT3 target genes are involved in upregulation of proliferation and downregulation of apoptosis, and have been implicated in the initiation of HCC. In hepatic inflammation, IL-6 secreted macrophages facilitated transformation of hepatocytes at early stages of hepato-carcinogenesis [86]. Thus, activated IL6/STAT3 signaling pathway in fibrotic liver due to chronic inflammation may render the liver prone to develop HCC. Of note, IL6/STAT3 can crosstalk with NF-κB signaling in inducing inflammation and liver cancer [83].

### 4.3. Insulin-Like Growth Factors (IGFs)

In mammals, IGFs play important roles in various cellular processes in the liver including cell growth, proliferation, and differentiation, as well as tissue repair and hepatic pathogenesis [87]. IGFs and their receptors (IGF-1R, IGF-2R) also regulate metabolic processes such as lipogenesis, and glycogen storage in the liver [88]. Although liver synthesizes IGF-1, IGF-2 and their binding proteins (IGFBPs) at a high level under the normal condition, the expression levels significantly decrease under pathogenic conditions like non-alcoholic fatty liver disease (NAFLD), cirrhosis, and HCC [88].

Experimental studies have demonstrated the roles of IGF-1 in the suppression of liver fibrosis and hepato-carcinogenesis [89]. IGF-1 reduces lipogenesis in hepatocytes and inactivates hepatic stellate cells, therefore ameliorating fibrosis with decreased serum AST and ALT levels [89]. In murine models of NASH and cirrhosis, administration of IGF-1 consistently improved steatosis, inflammation, and fibrosis with inactivation of HSC [90]. In a carbon tetrachloride (CCl_4_)–treated cirrhotic model, ectopic expression of IGF-1 reduced fibrogenesis [91]. Although studies have shown that decreased levels of IGF-1 are associated with HCC development, there are lines of evidence showing tumor-promoting effects of IGF-1. IGFs can activate downstream RAS and mitogen-activated protein kinase (MAPK) signaling pathway which promotes cell proliferation and survival. In human HCC cell lines, IGF-1 has a positive effect on HCC growth and metastasis [92] and it was found that abundant IGF-1 was associated with risk of HCC development [93]. 

### 4.4. Platelet-Derived Growth Factor (PDGF)

PDGF is one of the key factors involved in hepatic fibrogenesis. Increased expression of PDGF is detected in rodent livers after liver injury and in human livers with cirrhosis [94,95,96]. Under physiological conditions, PDGF is mainly secreted by Kupffer cells. However, when tissue is damaged, a variety of stromal cells including fibroblasts and vascular endothelial cells can synthesize and secrete PDGF through autocrine and paracrine manners [97,98]. Members of the PDGF ligand family consist of 4 polypeptides, PDGF-A, PDGF-B, PDGF-C, and PDGF-D [99]. The function of the PDGF signaling is mediated through platelet-derived growth factor receptors (PDGFR), tyrosine kinase receptors, that are activated by PDGFs [100]. Binding of PDGFs to PDGFR triggers activation of RAS, leading to downstream activation of the Raf-1 and MAPK signaling cascade as well as phosphatidylinositide 3-kinases (PI3K) and the AKT signaling pathway [101]. Accordingly, PDGF signaling can affect a variety of cellular functions including cell growth, proliferation, and differentiation.

Campbell et al. reported that transgenic mice expressing PDGF-C developed liver fibrosis via the activation and proliferation of HCSs [102]. Of note, persistent expression of PDGF-C in the liver for more than 9 months led to the development of hepatocellular adenomas and carcinomas. Likewise, Maass et al. observed liver fibrosis in the liver of transgenic mice expressing PDGF-B [103]. Interestingly, spontaneous tumor development was rarely observed in the liver expressing PDGF-B. However, when the mice were treated with diethylnitrosamine and phenobarbital, a method for chemically induced liver carcinogenesis, the development of dysplastic lesions and their malignant transformation to HCC were significantly increased in PDGF-B transgenic mice, demonstrating a pro-tumorigenic role of PDGF in HCC development [103]. Furthermore, PDGFR-α was found to be up-regulated in early HCC when compared with dysplastic nodules [104], and an increase in PDGFR-α expression level was found in 64% cases (14/22) of HCC patients when compared with adjacent non-tumoral parenchyma [105]. Considering PDGF can promote both hepatic fibrosis and cancer, it is tempting to consider the signaling to be an important mechanistic link between the two pathological conditions in the liver. 

### 4.5. Sonic Hedgehog (SHH)

Hedgehog (HH) signaling regulates various cellular processes including proliferation, apoptosis, migration, and differentiation [106]. The pathway plays a pivotal role in tissue morphogenesis during fetal development as well. In particular, HH signaling modulates wound-healing responses in a number of adult tissues, including the liver [107]. The pathway is activated when ligands such as SHH bind to Patched (Ptch) receptors, leading to the release of Ptch-mediated repression of G protein-coupled receptor, Smoothened (Smo). Smo can then subsequently induce the activation and nuclear accumulation of Glioblastoma (Gli) transcription factors, target genes of which are related to growth, repair, inflammation, and etc. [106]. The HH signaling pathway is found frequently activated in various types of liver injury [108].

Association of HH signaling with fibrosis has been found in fibrotic human livers and various animal models of liver fibrosis. In patients with NAFLD and NASH, a strong correlation was found between the stage of hepatic fibrosis and the degree of HH activation (determined by nuclear accumulation of Gli transcription factors) [109]. Animal models of liver fibrosis induced by various methods, such as genetic ablation of Mdr2 or Ikkβ deletion, bile duct ligation, and a methionine and choline–deficient (MCD) diet, all showed activation of SHH signaling in livers [110,111,112]. 

Synthesis of SHH is stimulated by diverse factors that can induce liver damage. SHH molecules that are released from wounded hepatocytes engage receptors on HH responsive cells, which include hepatic stellate cells, sinusoidal endothelial cells and hepatic immune cells [113]. Activation of HH signaling in the stromal cells induces various changes in the microenvironment required for liver regeneration such as growth of liver progenitor populations, tissue remodeling, angiogenesis, and hepatocyte regeneration. However, excessive and persistent activation of HH signaling sometimes overrides successful regeneration of damaged liver and contributes to pathogenesis toward liver fibrosis and cirrhosis [109,114]. A transgenic mice model ectopically expressing SHH in the liver revealed hepatic fibrosis, signifying the role of SHH signaling in fibrogenesis in the organ [5]. Secretion of SHH from hepatocytes in the model activated HH signaling in HH-responsive cells such as cholangiocytes, endothelial cells, as well as HSC. Further, activation of HH signaling in the liver induced epithelial to mesenchymal transition [108]. As HH signaling is activated in liver, expression of myofibroblast-associated genes gradually increases [108], along with the accumulation of myofibroblasts [114]. 

Several recent papers have demonstrated that HH signaling can significantly contribute to the initiation and promotion of hepatic cancer. Sicklick et al. reported that HH signaling was found elevated in human HCC [115], and Eichenmuller et al. showed that blocking the HH signaling pathway with an antagonist, cyclopamine suppressed cell proliferation of hepatoblastoma [116]. Moreover, SHH expression in liver promoted tumor progression by inducing the transition from hepatocellular adenoma to HCC [6]. It is noteworthy that an elevated HH signaling is found in approximately 60% of human HCC [112,115,117,118]. As HH activation is widely observed in patients with liver fibrosis induced by NASH, the signaling might contribute to a high incidence of HCC in cirrhotic livers of NASH patients.

### 4.6. Tumor Growth Factor Beta 1 (TGF-β1)

TGF-β signaling regulates a wide variety of cellular processes, including apoptosis of hepatocytes, activation and recruitment of inflammatory cells into injured liver, and activation of quiescent HSCs making them give rise to collagen-producing myofibroblasts [119,120]. Among the TGF-β ligands (β1, β2, and β3) TGF- β1 is linked to hepatic fibrogenesis [121]. TGF-β1 is biologically active as s 25kDa homo-dimer linked by disulfide bonds. Receptors for TGF-β1 are present on virtually all cells, suggesting that ubiquitous distribution of its target cells in tissue. 

TGF-β signaling is highly involved in hepatic fibrosis, and has been known as a mater regulator of tissue fibrosis [122,123]. TGF-β signaling regulates various biological responses related to liver fibrosis including hepatic apoptosis, activation of HSC, tissue remodeling, etc. [124,125]. Ironically, TGF-β is known as a major tumor-suppressive signaling pathway that inhibits cell division and promotes apoptosis. Under certain cellular and genetic circumstances, however, TGF-β signaling can act as a tumor promoter via activating various oncogenic signaling pathways. For example, through non-canonical TGF-β signaling pathways, the signaling promotes phosphoinositide 3-kinase and various mitogen-activated protein kinase (MAP kinases) [126,127]. As well, under genetic context of p53 loss [128], YAP activation [129] and Tak1 deletion [130], TGF-β signaling is required for and/or promote tumorigenesis in the liver. Although it is still not understood how TGF-β signaling can be a tumor promoter during early stages of hepatic tumorigenesis, considering its pro-apoptotic and anti-proliferative functions, recent studies suggest that Snail, an EMT inducer and a TGF-β target, can play a pro-tumorigenic role in the liver [131,132,133] likely via promoting cellular proliferation.

## 5. Gas6/TAM Pathway in Liver Fibrosis and Cancer

Growth arrest-specific gene 6 (Gas6) product is a vitamin K-dependent protein [134] that activates a family of TAM (Tyro3, Axl, MERTK) receptors with tyrosine kinase activity [135]. TAM signaling plays a role in tissue development and homeostasis, and disposes of apoptotic cells [136]. The ligand for TAM receptors, Gas6 is overexpressed and secreted in response to both acute and chronic liver injuries [136]. 

In normal liver, Gas6 is mainly expressed in Kupffer cells while Axl, which is related to cell differentiation and carcinogenesis among TAM receptors, is expressed in macrophages and quiescent HSC [135]. MERTK is expressed in Kupffer cells and sinusoidal endothelial cells, but not in hepatocytes, while Tyro3 is only found in resident macrophages [137]. Upon liver injury, Gas6 is overexpressed in Kupffer cells and HSC, which promotes infiltration of monocytes into injured tissue areas [138]. Of note, serum Gas6 levels were high in patients with advanced fibrosis and cirrhosis [139,140]. In line with the findings, experimental murine models showed that increased Gas6 levels led to activation and proliferation of HSC via AKT phosphorylation and NF-κB activation, and contributed to fibrogenesis in the liver [138,140]. 

Numerous studies have shown that upregulation of Gas6/TAM can promote development of multiple types of cancer, including lung and gastric cancer [141]. The Gas6/TAM pathway can exert multiple pro-tumorigenic effects both on tumor cells and stromal cells [142]. Activation of Gas6/TAM signaling in cancer cells promotes their proliferation and inhibits their apoptosis while the signaling pathway can lead to a tumor-promoting microenvironment, for example, suppressing anti-tumor effects by natural killer (NK) cells. As more experimental and clinical studies are performed to reveal the hepato-carcinogenic roles of Gas6 and TAM, they are expected to uncover a mechanistic link between liver fibrosis and HCC through the Gas6/TAM signaling pathway.

## 6. Hippo-YAP/TAZ Signaling in Liver Fibrosis and Cancer

Hippo-YAP/TAZ signaling is activated by a variety of mechanical signals such as cell shape and ECM stiffness as well as cell–cell interactions, and transduces cell-specific transcriptional programs. Of note, the signaling is not only activated by the interaction between specific extracellular ligands and their cellular receptors, but is also regulated by modulation of cell adhesion and cell polarity [143,144,145,146]. Hippo-YAP/TAZ signaling has a major role in the regulation of cell proliferation, apoptosis, migration and differentiation, all essential for both developmental processes and homeostasis in adult organs [143]. Disruption of the Hippo signaling, or abnormal activation of Yes-associated protein (YAP; also known as YAP1) and transcriptional co-activator with PDZ-binding motif (TAZ; also known as WWTR1) leads to a number of diseases including inflammation, fibrosis and cancer [147]. 

The Hippo-YAP/TAZ signaling pathway consists of kinase cascades, mammalian sterile 20-like kinase 1 (MST1; also known as STK4) and MST2 (also known as STK3), large tumor suppressor kinase 1 (LATS1) and LATS2, the adaptor proteins Salvador 1 (SAV1), MOB1A and MOB1B, and YAP/TAZ [148,149,150]. YAP and TAZ proteins are inactivated by phosphorylation through the core Mst1/2-Lats1/2 kinase cascade. Phosphorylation of YAP/TAZ leads to cytoplasmic retention of the proteins via the interaction with 14-3-3 proteins and degradation via the ubiquitin-proteosome pathway [151]. When YAP/TAZ are dephosphorylated, they can translocate into the nucleus and activate transcription of their target genes through the interaction with the TEAD transcription factors (TEAD1–TEAD4) [152].

Hippo-YAP/TAZ signaling regulates the organ size and closely related to liver regeneration as demonstrated in animal models in which activation of YAP/TAZ promoted regeneration of the liver. Knockdown of MST1/MST2 using siRNAs or a pharmacological inhibitor targeting MST1/MST2, which led to dephosphorylation and subsequent nuclear accumulation of YAP/TAZ, augmented hepatocyte proliferation and liver regeneration after partial hepatectomy in mice [153,154]. A recent study also confirmed the role of YAP/TAZ during liver regeneration after ischemia-reperfusion (I/R). The proliferation and expansion of HSCs were prominent during liver recovery after I/R injury, in which the Hippo pathway was inactivated and YAP/TAZ was activated in HSCs. In addition, inhibition of YAP/TAZ activation by a chemical inhibitor attenuated proliferation of both hepatocytes and HSC [155]. Furthermore, YAP can function as a stress sensor, leading to the elimination of damaged hepatocytes [156]. YAP activation in damaged hepatocytes led them to migrate into the hepatic sinusoids and undergo apoptosis. In contrast, YAP activation in undamaged hepatocytes promoted cellular proliferation [156].

### 6.1. Hepatic Fibrosis

YAP/TAZ activation can promote hepatic fibrosis through the activation of HSCs in response to chronic liver damage [157,158]. A remarkable accumulation of nuclear YAP/TAZ was found in myofibroblasts and HSCs of human and mouse livers with fibrosis [157]. Activated YAP/TAZ upregulate ECM deposition and tissue stiffness, which facilitate fibrogenic processes [159]. In a mouse model of hepatic fibrosis induced by carbon tetrachloride (CCl_4_) administration, YAP translocated from the cytoplasm into the nucleus of HSCs, and increased expression of its target genes. Notably, treatment with pharmacological inhibitors of YAP suppressed HSC activation and hepatic fibrogenesis, indicating that YAP activation is essential for liver fibrosis in mice [157]. Also, Zhang et al. found that YAP/TAZ were over-expressed in fibrotic livers of mice treated with CCl_4_, and that YAP/TAZ degradation by omega-3 polyunsaturated fatty acids (ω-3 PUFAs) led to down-regulation of pro-fibrogenic genes in activated HSCs and fibrotic liver [160]. Furthermore, the level of TAZ expression in hepatocytes was elevated in a murine model of NASH, and the silencing of TAZ in the NASH model prevented or reversed inflammation, hepatocyte death, and hepatic fibrosis although there was no significant changes in the degree of steatosis. Of note, hepatocyte-targeted expression of TAZ in the NASH model promoted NASH features via the activation of the HH signaling pathway [161].

### 6.2. Liver Cancer

YAP/TAZ signaling is involved in multiple facets of carcinogenesis, including promotion of cellular proliferation, induction of tissue invasion of tumor cells, and maintenance of cancer stem cells (CSCs) [148,162,163,164]. Persistent upregulation of YAP/TAZ activity is capable of initiating tumorigenesis in the liver [162,163]. The signaling also significantly contributes to chemoresistance, metastasis, and the recurrence of cancer [162]. Increased cell survival mediated by repression of apoptosis is also a consequence of activated YAP/TAZ [165]. The connective tissue growth factor (CTGF) and extracellular matrix protein CCN1 (CYR61), which are targets of YAP/TAZ, have been reported to inhibit apoptosis in liver cells [166,167]. Additional mechanisms also contribute to repression of apoptosis by YAP/TAZ, including up-regulation of pro-survival factors such as B cell lymphoma 2 (BCL 2) family members [168]. 

Of note, YAP/TAZ can promote tumorigenesis via cross-talks with diverse oncogenic signaling pathways [169,170,171,172]. Increased ectopic expression of YAP in an immortalized human hepatocyte cell line confers tumorigenic potentials via AXL, a receptor tyrosine kinase, as a major downstream factor [173]. A NUAK family SNF1-like kinase 2 (NUAK2) also known as SNF1/AMP kinase-related kinase (SNARK) participates in a positive feedback loop to maximize YAP activity through promotion of actin polymerization and myosin activity. The pharmacological inactivation of NUAK2 inhibits YAP-dependent cancer cell proliferation. These results demonstrate the role of kinase NUAK2 as a mediator of YAP-driven tumorigenesis [174]. Furthermore, it was reported that YAP expression reduced cellular senescence while silencing of YAP inhibited cell proliferation and induced premature senescence [175]. In line with the pro-tumorigenic functions, activation of YAP/TAZ was found at a high frequency in liver cancer and significantly correlated with poor prognosis [176,177,178].

Several lines of research using genetically engineered mouse models indicate that the Hippo-YAP/TAZ signaling pathway can induce cancer initiation and progression. Liver-specific deletion of both MST1 and MST2, leading to a subsequent activation of YAP/TAZ, was found to induce liver enlargement in young adult mice due to uncontrolled cell proliferation. MST1/2 ablated livers in the mice eventually developed liver cancers exhibiting either HCC or mixed hepatocellular and cholangiocellular carcinoma (mixed HCC-CCA) [179,180]. Similarly, transgenic mice expressing YAP showed enlarged livers after 8 weeks of YAP induction, and later on exhibited a number of nodules throughout the hepatic parenchyma [181]. 

The significance of YAP/TAZ overexpression in liver cancer was also investigated in HCC patients. In 177 patients with HCC, YAP overexpression was detected in 62% of tumor tissues, most of which exhibited nuclear accumulation of YAP in tumor cells. In addition, overexpression of YAP in tumor cells was significantly associated with poorer differentiation and elevated levels of serum α-fetoprotein (AFP) [182].

### 6.3. YAP/TAZ Linking Hepatic Fibrosis and Cancer

**Figure 2:**
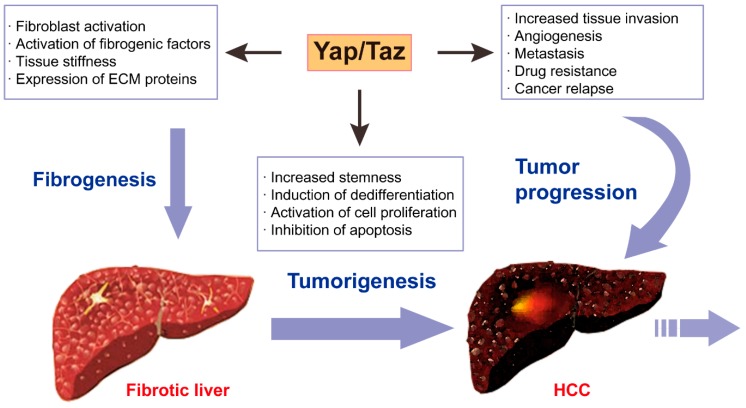
Schematic illustration of the roles of YAP/TAZ signaling in hepatic fibrosis and cancer.

The Hippo signaling pathway and its downstream effectors, YAP/TAZ have a strong correlation with hepatic fibrogenesis, and are critical regulators of hepatic tumorigenesis (Figure 2). The Hippo-YAP/TAZ signaling pathway also exerts significant effects on tumor microenvironment by maintaining cancer-associated fibroblasts (CAFs) and promoting neo-angiogenesis [147,157,183]. Moreover, in liver-specific conditional knockout mice, deletion of MST1/2 and SAV1 induced inflammation and elevated expression of pro-inflammatory cytokines such as IL-6 and TNF-α [179,184]. As well, it was recently reported that YAP/TAZ promoted liver inflammation and liver cancer. In hepatocytes with genetic deletion of Mst1/2, monocyte chemoattractant protein-1 (Mcp1) expression was highly up-regulated which led to massive infiltration of macrophages. In addition, macrophage ablation or Mcp1 deletion in the Mst1/2 knockout mice showed reduced hepatic inflammation and HCC development, whereas Yap elimination abolished the induction of Mcp1 expression and restored normal liver growth [185]. Another study found a strong correlation between TAZ expression in human liver tumors and secretion of pro-tumorigenic inflammatory cytokines such as IL-6 and C-X-C motif chemokine ligand 1 (Cxcl1) [186]. As hepatic fibrosis is mainly induced by chronic inflammation in the liver, YAP/TAZ might be a strong promoter for both hepatic fibrosis and liver cancer.

## 7. Conclusions

Liver fibrosis and cirrhosis have long been regarded as major risk factors for HCC. Fibrotic livers establish a pro-tumorigenic microenvironment via an increase in genetic alterations and chromosomal instabilities, as well as activation of various oncogenic signaling pathways. Recent studies have found YAP/TAZ signaling acting as a major mechanistic link between liver fibrosis and HCC. Further study in this field is needed to better understand the pathogenic process toward liver cancer and to prevent the development of HCC in cirrhotic background, considering that there are currently no effective therapies for HCC.
